# Fabrication of Thermo-Responsive Controllable Shape-Changing Hydrogel

**DOI:** 10.3390/gels8090531

**Published:** 2022-08-25

**Authors:** Yi Luo, Werner Pauer, Gerrit A. Luinstra

**Affiliations:** Institut für Technische und Makromolekulare Chemie, Universität Hamburg, 20146 Hamburg, Germany

**Keywords:** hydrogel, double network, thermo-responsive, 3D printed model shape

## Abstract

Temperature response double network (DN) hydrogels comprising a network formed by polymerization of methacrylic acid (MA) modified PVA, N,N’-methylene bis(acrylamide), N-isopropylacryl amide (NIPAM), and one formed from crystalline polyvinyl alcohol (PVA) are prepared in a 3D printed tailor-made mold. The (PVA-MA)-g-PNIPAAm thermoset intermediate is formed in water by a radical, photo-initiated process, and in the presence of dissolved PVA polymers. A subsequent freezing-thawing sequence induces the crystallization of the PVA network, which forms a second network inside the thermoset NIPAM polymer. The prepared hydrogel is thermoresponsive by the phase transition of PNIPAAm segments (T ≈ 32 °C) and has good mechanical properties (tensile strength 1.23 MPa, compressive strength 1.47 MPa). Thermal cycling between room temperature at 40 or 50 °C shows the product converses from a virgin-state to a steady-state, which most likely involves the reorganization of PVA crystals. The swelling-deswelling cycles remain clear at a length change of about 13%.

## 1. Introduction

Hydrogels are 3D-polymeric network materials that have the ability to immobilize a larger amount of water molecules [1,2,3]. One current area of study in this context is the development of stimuli-responsive hydrogels. The interest results from their “smartness” in terms of deformation in response to external stimuli, such as heat [4,5], near-infrared light [6,7], pH [8,9], electricity [10,11], chemicals [12,13], and also by multiple stimuli at once [14,15,16,17]. Thermo-responsivity has gained the most attention, perhaps because temperature is most easily varied. Poly-N-isopropyl acrylamide (PNIPAAm) is one of the most favored representatives of a temperature-responsive polymer. Thermal modulation reversibly switches the polymer through the volume phase transition temperature (VPTT) around 33–35 °C from a water-swollen, hydrophilic state into a de-swollen, hydrophobic state. [18,19,20] PNIPAAm hydrogels were applied in various fields with soft actuator actions, such as analytical separation and detection [21], antifouling coatings [22], soft robotics [23,24], microfluidic flow controlling [25,26], and also in additive manufacturing (AM) [27,28,29]. PNIPAAm hydrogels, however, have a limited efficiency as an actuator because of their limited mechanical property profile. The low solid content (70–98% water content) of the gel and absent energy dissipating mechanisms lead to low strength and fragility [30]. The property profile hampers their potential application as a temperature programmable unit.

Various kinds of strategies have been followed to improve hydrogel mechanical properties, involving structural variations [31,32] or the introduction of discrete fillers [33,34,35]. A toughening of hydrogels can also be achieved through the construction of a double network (DN), which normally is composed of an asymmetrical interpenetrating network (IPNs) [36,37,38,39]. The first DN-hydrogels comprised a charged, densely crosslinked primary network of poly(2-acylamide-2-methyl-propane sulfonate acid) and a neutral, loosely crosslinked second network of poly(acrylamide) (PAAM) [40]. Otherwise, only a few kinds of DN thermo-responsive hydrogels with PNIPAAm appear to have been reported.

The concept of incorporating the responsiveness of PNIPAAm in a DN (double network) hydrogel, however, seemed one with more potential for obtaining a mechanically robust attenuator. The combination of a NIPAM-based polymer with a further hydrogel-forming polymer in the form of polyvinyl alcohol (PVA) was chosen as this further approach. PVA has been widely used for the fabrication of hydrogels with more enhanced mechanical properties, in particular with respect to fracture toughness [41,42]. The option of forming nanocrystalline domains from PVA provides the potential to build a double network, i.e., next to one involving a PNIPAAm moiety. The NIPAM-based hydrogel in this study was prepared in a multistep procedure, comprising the preparation of methacrylate functionalized PVA and copolymerization with NIPAM using photoinitiation in a 3D printed mold. Photo-crosslinking technology realized by low-energy ultraviolet light is a simple, fast, green, and low-cost approach for hydrogel fabrication. The properties of the products were mapped and related to the various protocols of synthesis. Generally, the hydrogel showed high responsiveness to temperature, appeared tough, and is readily programmable. This kind of gels may hold substantial potential for application in the construction of soft robots and programming actuators.

## 2. Results and Discussion

### 2.1. Hydrogel Preparation and Structure

**Scheme 1:**
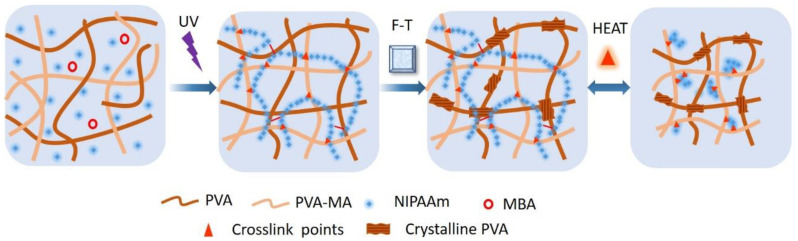
The forming and thermo-responsiveness mechanism of DN hydrogels.

**Figure 1:**
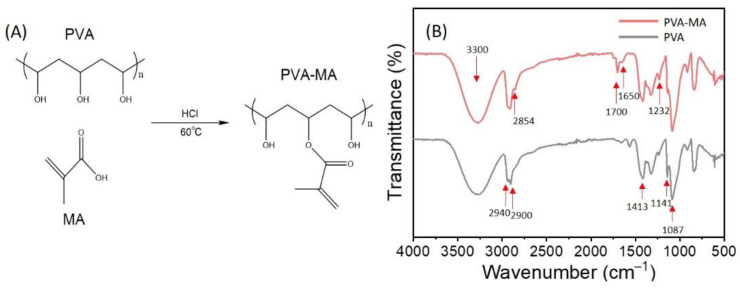
(**A**) Synthesis route of PVA-MA from poly (vinyl alcohol) (PVA) and methacrylic acid (MA). (**B**) FT-IR spectra.

The synthesis of the double network of PVA/(PVA-MA)-g-PNIPAAm proceeds in a sequence of several steps (Scheme 1). The first step is the condensation of methacrylic acid (MA) to PVA for obtaining a PVA precursor with a radically addressable vinylic side chain. FT-IR and NMR analysis of the PVA-MA product is consistent with the expectation of olefinic ester formation (Figure 1 and Figure S1). The presence of characteristic absorbances of olefinic entities at 2854 and 1650 cm^−1^ and ester entities at 1700 and 1232 cm^−1^ is taken as evidence.

The resulting functionalized PVA-product was next mixed with a further smaller aliquot of PVA (5 and 10 wt%) to reach a blend of constitution PVAx/PVA-MA (x = 5, 10). A PVA-MA-containing network was subsequently formed by copolymerizing NIPAM and N,N’-methylene bisacryl amide (BIS), and the MA entities of the modified PVA. The physical integration of PVA into the crosslinked PVA-MA, NIPAM monomer, and BIS terpolymer gives a 3D network with properties reminiscent of its components.

**Figure 2:**
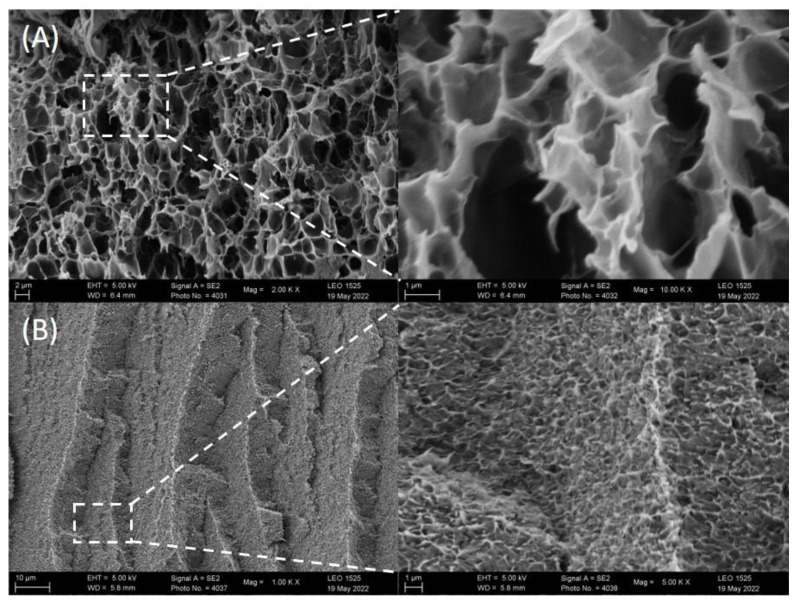
SEM images of the DN hydrogels with 10 wt% PVA before (**A**), and after (**B**) F-T operation.

The presence of the (unfunctionalized) PVA polymer provides the option of forming a second network by nano-crystallization, strengthening the primary responsive hydrogel based on NIPAM. The partial crystallization of PVA can be induced by a “freeze-thaw” (F-T) sequence, i.e., by cooling the hydrogel below the freezing point of the water. The originally meta-stabile dissolved PVA crystallizes during the procedure inside the hydrogel. Subsequent thawing gives the DN hydrogel of (i) crosslinked PNIPAAM-PVA-BIS and (ii) nanocrystalline PVA. The crystallization is readily recognized in the change from a course to a much finer structured morphology of the hydrogel (Figure 2).

The PVA crystallites have a melting point of 95 °C, giving the maximum temperature for application without loss of properties well below that temperature. The functionalized PVA-MA itself does not crystallize anymore. The hydrogel is transparent. Thus, 3D printing technology can be employed for diversiform units’ fabrication using photo-initiators and also for more complex items.

### 2.2. Mechanical Properties

**Figure 3:**
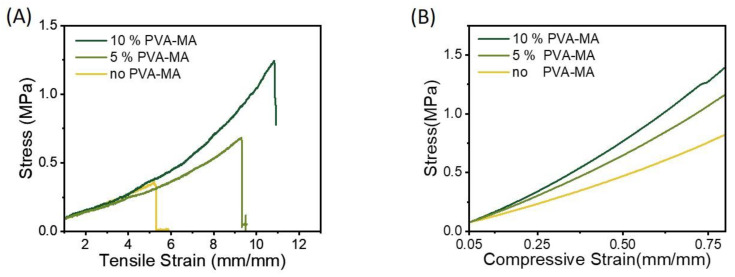
Tensile (**A**) and compressive (**B**) stress–strain curves of PVAx/(PVA-MA)-g-PNIPAM (x = 0, 5, 10) hydrogels.

Typical successive tensile and compressive stress responses of crosslinked hydrogels were obtained (Figure 3). The mechanical properties of the PVAx/(PVA-MA)-g-PNIPAAm hydrogels give the highest values of stress (1.25 MPa) and elongation at break (10%) for samples with the largest crystal content, i.e., with 10% of PVA. Samples with 5 wt% of PVA already exceed the tensile strength of the hydrogels without PVA by almost a factor of two (0.68 vs. 0.36 MPa stress at break). A similar trend can also be observed for the mechanical behavior on compression (1.47 vs. 0.87 MPa at a strain of 75% and rate of 10 mm/min).

### 2.3. Swelling Properties

**Figure 4:**
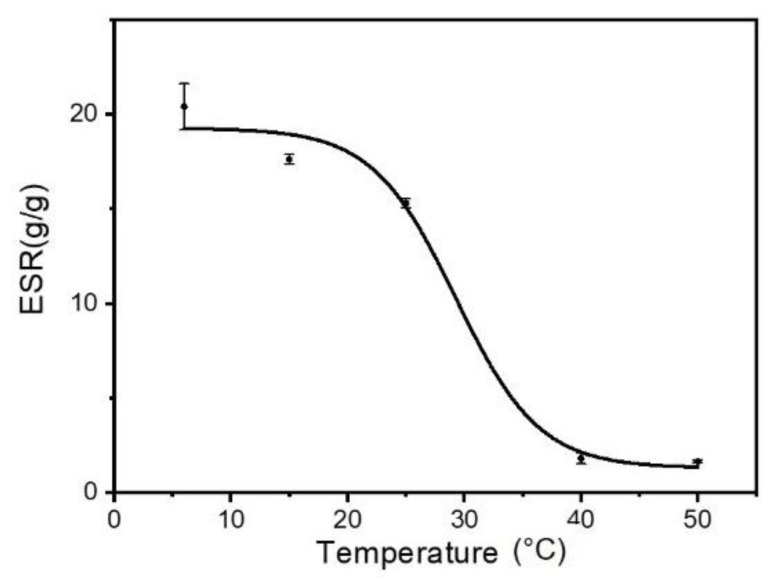
Equilibrium swelling ratio (ESR) of PVA10/(PVA-MA)-g-PNIPAM hydrogel in the temperature range of 6 to 50 °C.

The physical network formed by the crystals of PVA in the hydrogel gave a more compact hydrogel, which also impacts the responsiveness to thermal changes (as is expected). A hydrogel with a higher than 10 wt% of PVA content was considered unfavorable (too stiff). Further experimentation was therefore conducted with the strongest gel containing 10 wt% of PVA (PVA10/(PVA-MA)-g-PNIPAAm). The equilibrium swelling ratio of the hydrogel PVA10/(PVA-MA)-g-PNIPAAm reduced substantially from 18.6 to 1.2 in the range between 6 and 50 °C (Figure 4) [43].

**Figure 5:**
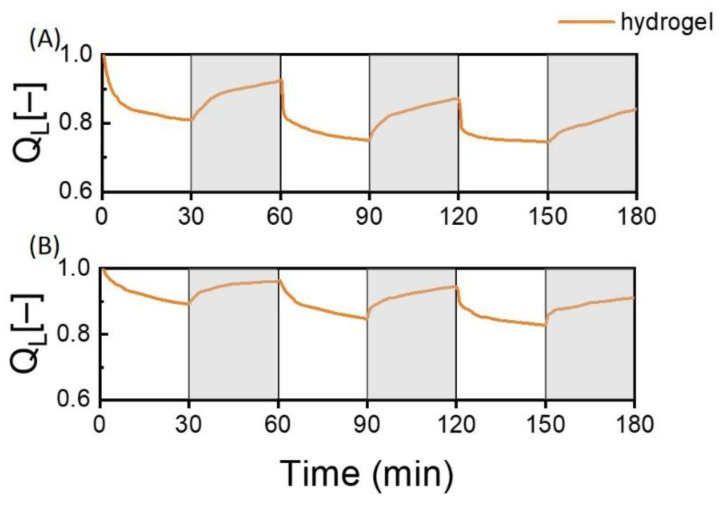
Length normalized swelling degrees (Q_L_; cf 4.4.3) of PVA10/(PVA-MA)-g-PNIPAAm on cycling between 25 °C and 50 °C (**A**) or 40 °C (**B**) in deionized water.

The response of the gel to a sudden cooling from 50 °C is quite fast (Figure 5). A major part of the shrinkage takes place within a couple of minutes. The same measurements starting at 40 °C show a smaller and slower response. This is in accordance with the equilibrium swelling property; the swelling at 40 °C is not the maximum volume of the gel. The hydration of the de-swollen gels on subsequently heating is somewhat slower. Here, the major part of the response takes less than 10 minutes.

**Figure 6:**
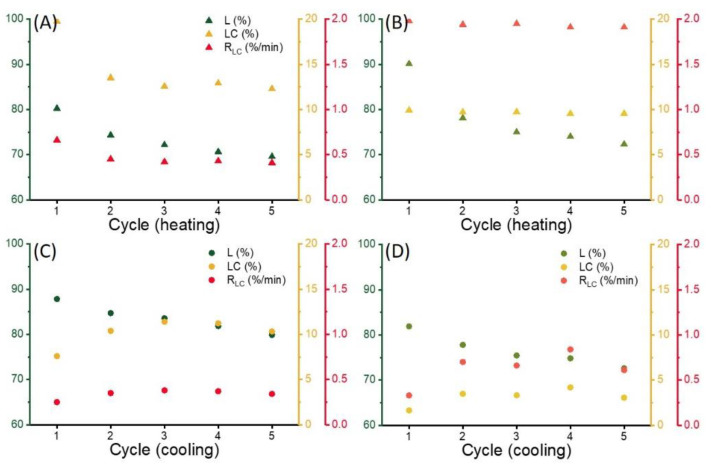
Length (L), final length change (LC) after 30 min, and its linear rate constant (R_LC_) of PVA10/(PVA-MA)-g -PNIPAAm while cycling in 30 min intervals between 25 °C and 50 °C in deionized water: (**A**) total response on heating with (**B**) response in the first 10 min, and (**C**) on cooling with (**D**) for first 10 min.

The hydrogel of PVA10/(PVA-MA)-g-PNIPAAm does not form obviously in the synthetic procedure in its most stable state. The analysis of the length normalized dynamic swelling degrees between 25 °C and 40 °C or 25 °C and 50 °C shows that the recovery after shrinkage is decreasing with the number of cycles (Figure 5, Table S1). Both the maximum length and the total response are deteriorating with the number of cycles. The largest difference is between the first and second full cycle (Figure 6) after which an equilibration process seems to set in, wherein 9% (40 °C) or 16% (50 °C) of the length is not recovered. A stable value was not yet reached after 5 full cycles (Figure 6), but the change of length seems to approach the value of around 13%.

The rate constant of the swelling-deswelling processes in the first minutes is not substantially changing during cycling (Figure 6B,D). The deswelling remains about twice as fast as the swelling. The fast part of the response may be related to the faster solvation of the NIPAM chain segments [28]. The fast part of the response is at 70% of the total change of length on cooling. The changes in length between full cycles may thus most likely be associated with a reorganization of the hydration of the PVA crystals or PVA segments. PVA hydrogels may contain two types of water molecules, one type directly interacting with the hydroxyls of the PVA (bound water) and free water [44]. Here, interactions of the PVA with the NIPAM segments may be envisioned. Each deswelling process of the 20 mm × 20 mm × 2 mm specimen would release a fraction of non-bound water, possibly changing the crystalline content and/or increasing the polymer–polymer segment interactions. This behavior is the topic of further investigations with the aim to stabilize the hydrogel against this loss of volume. This type of hydrogel exhibits potential applications as actuation units in terms of the robustness of the swelling-deswelling loops.

### 2.4. Tailored Shape Fabrication

**Figure 7:**
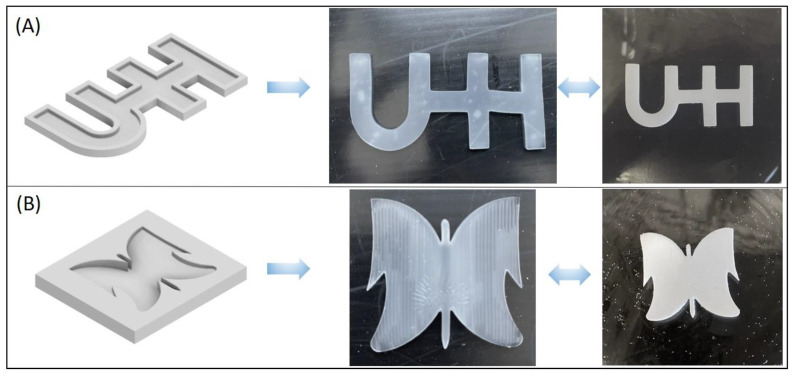
Multiverse shape of the hydrogel. (**A**) logo of UHH; (**B**) butterfly.

Fused Filament Fabrication (FFF) technology is employed here for the cast manufacture. The design within Inventor (Autodesk^®^ Inventor^®^ 2021, CADAC group, Frankfurt am Main, Germany) and printing in a 3D printer (Ultimaker 3) allows for preparing molds for a hydrogel soft system of many shapes. The components were photopolymerized and subsequently, the freezing-thawing cycle gave the free-standing DN hydrogels as an image. Thermo-responsive hydrogel in the shape of the logo of Hamburg university and a butterfly are fabricated successfully by this method (Figure 7 and Figure S2). The clear hydrogel units held their shape adequately in response to temperature changes.

## 3. Conclusions

An easy method is reported for the fabrication of thermo-sensitive double network PVA/(PVA-MA)-g-PNIPAAm hydrogel units of the desired shape by -FFF-3D -printing-technology-prepared form. The components for preparation, namely, PVA, MA, BIS, and NIPAAm are readily available, and simple esterification and photopolymerization are the only chemical steps. The hydrogel has enhanced mechanical properties over a (PVA-MA)-g-PNIPAAm hydrogel while keeping a length response after five cycles of about 13% (volume of 34%). The presented design would broaden the applications of hydrogel units in soft machines, actuators, and relevant fields.

## 4. Materials and Methods

### 4.1. Materials

Poly (vinyl alcohol) (PVA, 99+% hydrolyzed, Mw 85,000-124,000), hydroquinone, N, N’-methylenebis (acrylamide) 99%, Lithium phenyl-2,4,6-trimethyl-benzolphosphonate (TPO-Li), triethyl amine were obtained from Sigma-Aldrich (Taufkirchen, Germany). N-isopropyl acrylamide (NIPAM, 99%, pure, stabilized) was purchased from Acros Organic (Darmstadt, Germany). Methacrylic acid was obtained from Merck KGaA (Darmstadt, Germany). All reagents were used as received. Demineralized water (DI) was obtained from lab water systems.

### 4.2. Synthesis of PVA-MA

PVA (15 g) was combined with 135 mL of deionized water in a three-necked round flask equipped with a condenser. The flask was put in an oil bath of 90 °C, and the mixture was mechanically agitated for 1 h to give a clear solution. Subsequently, 30 mg of hydroquinone was added to the solution. After cooling to ambient temperature, 20 mL of MA and 7.5 mL of hydrochloric acid (0.5 M) were added to the solution. The resulting mixture was heated to 60 °C, and the esterification was carried out while stirring (at 300 rpm) overnight. After cooling down to room temperature, 0.5 mL of triethyl amine was added to end the reaction. The resulting solution was then diluted 10 times and precipitated in acetone (1.5 L). The precipitate was filtered, washed with acetone, and dried at 60 °C under a dynamic vacuum of 1000 mbar.

### 4.3. Hydrogel Preparation

PVA and PVA-MA were dissolved in deionized water at 90 °C by stirring for 1 h. After cooling to room temperature, an amount of NIPAM was added to the solution which was stirred to reach a homogenous mixture. Subsequently, a photo-initiator (TPO-Li) and crosslinking agent (N,N’-methylene bisacrylamide) (BIS) were added. The concentration of PVA-MA and PVA was both 10 wt%, whereas NIPAM was 20 wt%. The concentration of BIS and TPO-Li were at 0.3 wt% and 0.1 wt% with respect to 60 wt% DI water. The mixture was subjected to ultrasonic impact for 30 min to remove dissolved components of air.

**Figure 8:**
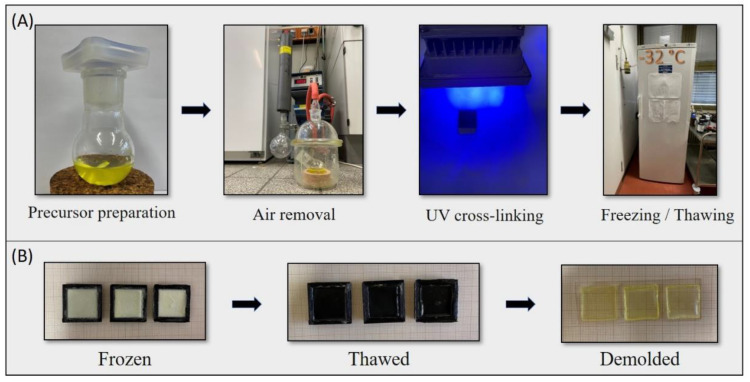
(**A**) Preparation processes of photo-crosslinked PVA/(PVA-MA)-g-PNIPAM hydrogels. (**B**) Photos of PVA/(PVA-MA)-g-PNIPAM hydrogel in processes.

Hydrogel formation was induced by site-specific photocuring of the aqueous precursor (Figure 8). The precursor solution was syringed into the desired mold formed by fused filament fabrication (FFF) using a 3D printer (Ultimaker 3, Ultimaker B.V., Framingham, MA, USA) from polylactide (PLA) filament. The mold was submerged in an ice water bath and exposed to a high-power UV light (405 nm, Geeetech, Shenzhen, China) for 30 min. The formed polymeric products were then de-molded, rinsed with DI water, and immersed in DI water for 24 h to extract soluble components (residual monomers, etc.). The obtained entities were subjected to freezing/thawing treatment (freezing at −32 °C overnight, thawing for 1 h at 25 °C). The unfrozen hydrogels were used for characterization and analysis.

### 4.4. Characterization

#### 4.4.1. FT-IR Characterization

The Fourier-transform infrared spectroscopy (FT-IR) analysis was conducted by Vertex 70 (BRUKER OPTIK GmbH, Ettlingen, Germany). The spectrum was recorded in the spectral range of 4000–400 cm^−1^ with a 2 cm^−1^ resolution and 32 scans.

#### 4.4.2. Equilibrium Swelling

The swelling properties of the hydrogels over the temperature range from 6 to 50 °C were measured gravimetrically using the samples from the 3D printed square model (2 × 2 cm). Equilibrium swelling ratio (ESR) was determined as the weight of fully water-swollen hydrogel (Ws) divided by the weight of the dry hydrogel (Wd). The equilibrium swelling ratio (ESR) was calculated as ESR= Ws/Wd∗100%. The swelling equilibrium state of hydrogel was reached by immersing the hydrogel units in DI water for 24 h at a designated temperature. The hydrogel samples were accurately weighted after wiping the water from the surface with filter paper. Subsequently, each sample was dried at 60 °C for 2 h under a dynamic vacuum of 1000 mbar and weighted for the dry weight.

#### 4.4.3. Length Normalized Swelling Degrees

Typical samples were equilibrated in DI water for 24 h at room temperature. Cycles of temperature changes in a range exceeding the LCST and room temperature were applied. The whole process of volume change was recorded by the camera and the dimensional changes data were analyzed using Imagine-Pro Plus 6.0 software (Media Cybernetics, Rockville, MD, USA). Length normalized swelling degrees Q_L_ were determined as the length of the swollen hydrogel (Ls) divided by the length of the equilibrium hydrogel L_Q_ ($Q_{L}=\frac{L_{S}}{L_{Q}}\cdot 100\%$).

#### 4.4.4. Dynamic Mechanical Analysis (DMA)

A Zwick Z2.5 universal testing machine (Zwick/Roell, Ulm, Germany) was used for measuring dynamic mechanical properties. The tensile modulus was evaluated in accordance with ASTM D412 (rate of elongation of 5 mm/min). Dumbbell-shaped specimens (w 4 mm, d 2 mm, L 25 mm) were prepared as exemplified above, now using an appropriate PLA model. The compressive modulus was performed along with DIN ISO 7743 [37]. The standard cylinder specimens (diameter of 17.8 mm, height of 25 mm) were prepared in a suitable 3D–printed PLA part. Four circles of compression and release were performed till a strain of 25% was achieved for use at a rate of 10 mm/min. The force–deformation curve was taken from the last circle. At least five replicates were taken and averaged.

## Data Availability

The datasets generated and analyzed for this study can be shared upon reasonable request to the corresponding author.

## Supplementary Materials

The following supporting information can be downloaded at: https://www.mdpi.com/article/10.3390/gels8090531/s1, Figure S1: NMR spectra of PVA-MA; Figure S2: Illustration of the 3d model. UHH logo (**A**) and Butterfly (**B**); Table S1: Length (L), final length change (LC), and its linear rate constant (RLC) of PVA10/(PVA-MA)-g -PNIPAAm while cycling in 30 min, first 5 min, and first 10 min intervals between 25 °C and 50 °C in deionized water.
