# Supplementary material for: Fabrication of Thermo-Responsive Controllable Shape-Changing Hydrogel
Source: Gels. 2022 Aug 25;8(9):531. doi: 10.3390/gels8090531 (PMC9498808; doi:10.3390/gels8090531)
Supplement: Supplementary file 1 [file gels-08-00531-s001.zip › gels-1822452-supplementary.pdf]

gels MDPI

*Supplementary Materials*

# Fabrication of Thermo-Responsive Controllable Shape-Changing Hydrogel

**Yi Luo, Werner Pauer and Gerrit A. Luinstra ***

Institut für Technische und Makromolekulare Chemie, Universität Hamburg, 20146 Hamburg, Germany
* Correspondence: luinstra@chemie.uni-hamburg.de

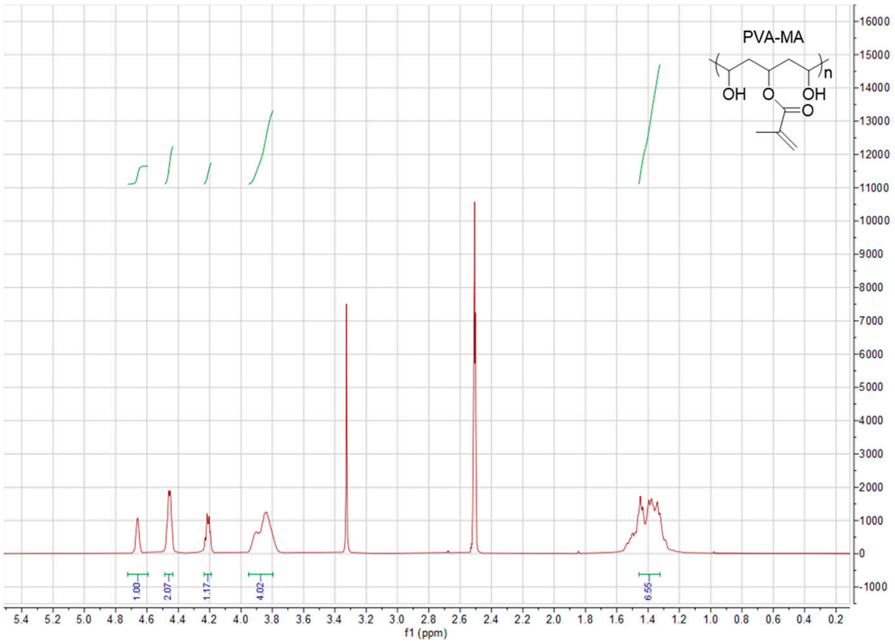


**Figure S1**. NMR spectra of PVA-MA.

**Table S1**. Length (L), final length change (LC), and its linear rate constant ($R_{LC}$) of PVA10/(PVA-MA)-g -PNIPAAm while cycling in 30 min, first 5 min, and first 10 min intervals between 25 °C and 50 °C in deionized water.

| | all 30 min | | | first 5 min | | | first 10 min | | |
|---|---|---|---|---|---|---|---|---|---|
| operation | L (%) | LC (%) | $R_{LC}$ (%/min) | L (%) | LC (%) | $R_{LC}$ (%/min) | L (%) | LC (%) | $R_{LC}$ (%/min) |
| heat 1 | 80,24 | 19,76 | 0,66 | 90,10 | 9,91 | 1,98 | 85,77 | 14,23 | 1,42 |
| cool 1 | 87,82 | 7,58 | 0,25 | 81,89 | 1,64 | 0,33 | 83,40 | 3,16 | 0,32 |
| heat 2 | 74,32 | 13,50 | 0,45 | 78,11 | 9,71 | 1,94 | 76,93 | 10,89 | 1,09 |
| cool 2 | 84,71 | 10,39 | 0,35 | 77,80 | 3,48 | 0,70 | 82,75 | 8,43 | 0,84 |
| heat 3 | 72,15 | 12,56 | 0,42 | 74,98 | 9,73 | 1,95 | 73,52 | 11,19 | 1,12 |
| cool 3 | 83,54 | 11,39 | 0,38 | 75,45 | 3,31 | 0,66 | 78,68 | 6,54 | 0,65 |
| heat 4 | 70,64 | 12,90 | 0,43 | 74,00 | 9,54 | 1,91 | 72,61 | 10,92 | 1,09 |
| cool 4 | 81,84 | 11,21 | 0,37 | 74,82 | 4,19 | 0,84 | 77,68 | 7,05 | 0,70 |
| heat 5 | 69,56 | 12,29 | 0,41 | 72,29 | 9,55 | 1,91 | 71,05 | 10,80 | 1,08 |
| cool 5 | 79,87 | 10,32 | 0,34 | 72,59 | 3,04 | 0,61 | 74,97 | 5,41 | 0,54 |

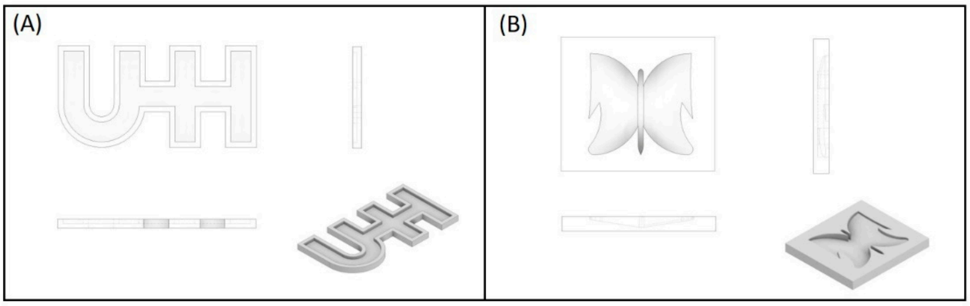


**Figure S2**. Illustration of the 3d model. UHH logo (A) and Butterfly (B).
